# The Endocannabinoid System and Eating Behaviours: a Review of the Current State of the Evidence

**DOI:** 10.1007/s13668-022-00436-x

**Published:** 2022-08-18

**Authors:** Nathaly Aguilera Vasquez, Daiva E. Nielsen

**Affiliations:** School of Human Nutrition, McGill University, Macdonald Campus, 21111 Lakeshore Rd, Ste. Anne-de-Bellevue, Quebec, H9X 3V9 Canada

**Keywords:** Endocannabinoid, Cannabinoid receptor, Eating behaviour, Hedonic eating, Food intake

## Abstract

**Purpose of the Review:**

The endocannabinoid system (ENS) has emerged as an important factor in food intake and may have implications for nutrition research. The objective of the current report is to summarise the available evidence on the ENS and eating behaviour from both animal and human studies.

**Recent Findings:**

The literature reviewed demonstrates a clear link between the ENS and eating behaviours. Overall, studies indicate that 2-arachidonoylglycerol (2-AG) and N-arachidonoylethanolamine (AEA) via cannabinoid receptor-1 (CNR1) binding may stimulate hunger and food intake while oleylethanolamide (OEA) may inhibit hunger. Mechanisms of these associations are not yet well understood, although the evidence suggests that there may be interactions with other physiological systems to consider. Most studies have been conducted in animal models, with few human studies available.

**Summary:**

Additional research is warranted among human populations into the ENS and eating behaviour. Evaluation of relationships between variation in ENS genes and dietary outcomes is an important area for investigation.

## Introduction

The endocannabinoid system (ENS) is a signalling system consisting of endocannabinoids (ECs), endogenous compounds derived from long-chain polyunsaturated fatty acids, and cannabinoid receptors which serve as binding sites for ECs to modulate various regulatory reactions in the body [1–3]. In 1990, in searching for a mechanism explaining the therapeutic effects of cannabis, Matsuda et al. isolated a first G-protein couple receptor that reacted with active compounds in cannabis in rat brain, cannabinoid receptor-1 (CNR1) [4]. This was quickly followed by the discovery of cannabinoid receptor-2 (CNR2) in 1993, not in rat brain as seen for CNR1, but in rat spleen [5]. This not only helped further the understanding of the binding of active cannabis compounds, but also paved the way to the discovery of ECs that bind these receptors, of which the first to be discovered were N-arachidonoylethanolamine (AEA) and 2-arachidonoylglycerol (2-AG), both derived from arachidonic acid [2]. Aside from the latter, which are the most common, there exist many other ECs including N-arachidonoyldopamine (NADA), 2-arichidonoylglycerylether (noladin ether) and O-arachidonoylethanolamine [6]. Evidence shows that AEA and 2-AG function through retrograde signalling where they are synthesised de novo based on intracellular calcium concentrations and activate CNR1 to inhibit neurotransmitter release [2, 7].

We now know that CNR1 is nearly ubiquitous in the human body, with expression in the central nervous system and many other tissues and systems (i.e. liver, reproductive system, gastrointestinal tract, skeletal muscles, the cardiovascular system) [7]. CNR2, on the other hand, is mainly expressed in immune cells and other peripheral tissues, but not in the central nervous system [7]. The ENS also contains endocannabinoid-like compounds—N-acylethanolamines (NEAs), e.g. palmitoylethanolamide (PEA), oleylethanolamide (OEA) and 2-monoacylglycerols (2-MAGs)—which are structurally similar to ECs but they do not bind to cannabinoid receptors, although they are metabolised through pathways involving the same enzymes [8, 9]. Nonetheless they play an important role in the ENS and have been linked to appetite and food intake [8]. Although the exact mechanisms of action of ECs and EC-like compounds are not yet fully understood, certain key enzymes have been highlighted such as N-acyl-phosphatidylethanolamines (NAPE)-hydrolysing phospholipase D involved in the synthesis of these compounds and fatty acid amide hydrolase (FAAH) involved in their degradation [9]. It is also recognised that ECs and EC-like compounds interact with receptors other than CNR1 and CNR2 which further contributes to the versatility of their biological functions [9].

Given the global rise in obesity and metabolic disorders, it is important to explore the various factors that may contribute to energy balance in humans. The ENS has emerged as an important actor in food intake and hedonic eating [10, 11]. Equally as important is understanding the role of genetic variation in the ENS and its impact on food behaviour and preference to identify vulnerable groups who may be most susceptible to hedonic eating and thus may need different diet therapy approaches for regulating eating behaviour. The objective of the current report is to summarise the available evidence on the ENS and eating behaviour from multiple modes of inquiry, including both animal and human studies.

## Methods

PubMed was searched for studies using keywords relating to the “endocannabinoid system” and “eating behaviour”. The search strategy focused on using “Medical subject headings” (MeSH terms) without language restriction as follows: (Endocannabinoid* [tiab] OR “endocannabinoid system” [tiab] OR cannabinoid* [tiab] OR “endocannabinoids” [MeSH Terms] OR “Cannabinoids” [MESH] OR “Cannabinoid Receptor Modulators” [MESH] OR “cannabinoid receptor agonists” [MeSH Terms]) AND (“eating behavior*” [tiab] OR “eating behaviour*” [tiab] OR appetite [tiab] OR “food intake” [tiab] OR “food consumption” [tiab] OR craving [tiab] OR “eating habit*” [tiab] OR “feeding behavior” [MeSH Terms] OR “feeding behavior” [tiab]). The search was run in January 2022 and was not restricted to any time period. Title and abstract screening was carried out in the first phase of inclusion, and this was followed by full-text screening. Additional articles were included through a search of reference lists of included studies. Screening was performed by NAV and DEN provided consultation to resolve uncertainties. Exclusion criteria included retrievals that were not primary research articles (e.g. review articles, perspectives, editorials), articles that did not consider eating behaviour, and articles that did not consider the ENS.

## Results

**Fig. 1:**
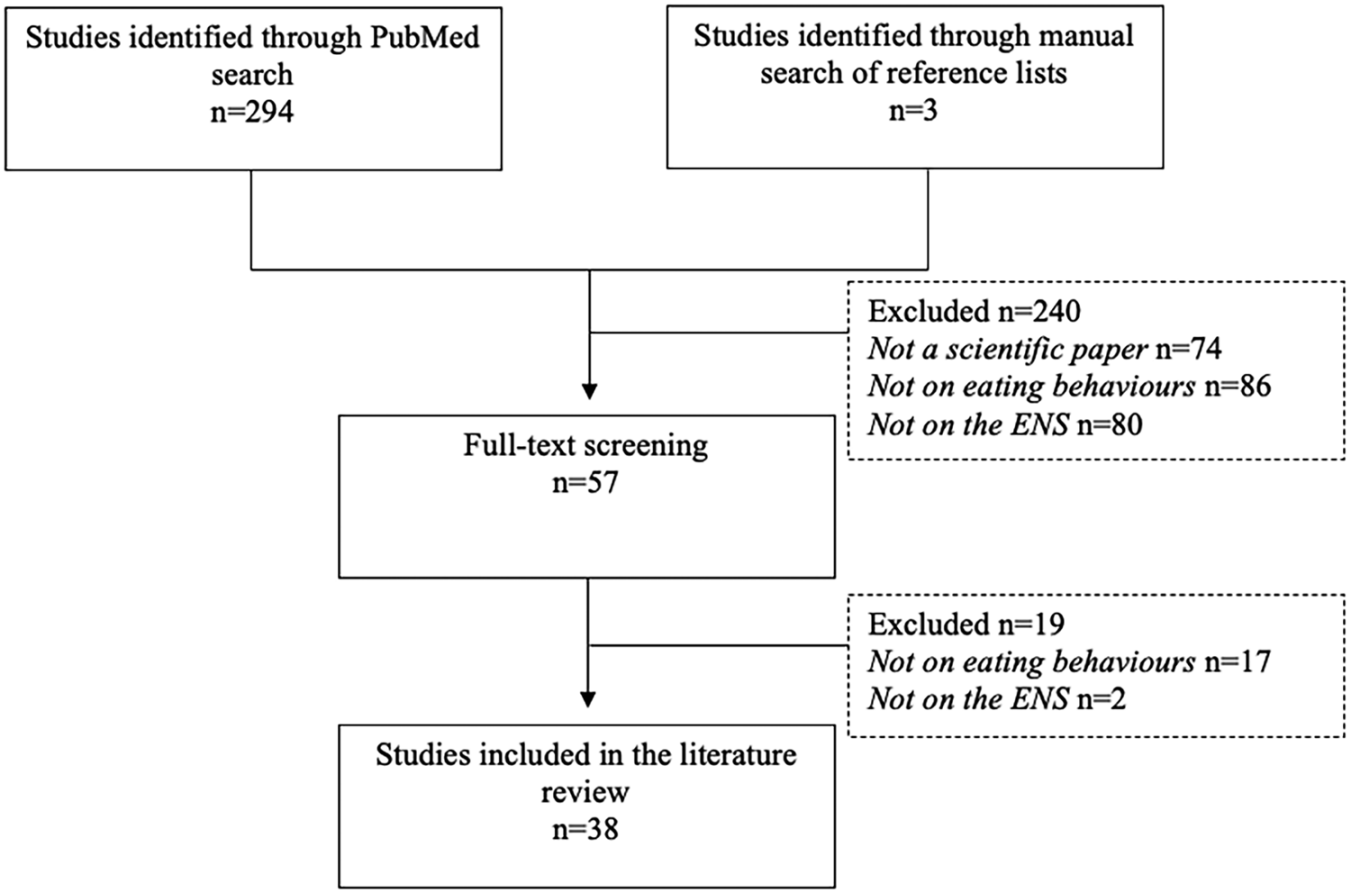
Summary of literature search and retrieved records

The search strategy retrieved a total of 294 articles and 3 articles were added from manual reference lists (Fig. 1). Upon completion of article screening, 38 articles were eligible to be included in the present review. Thirty studies were conducted in animal models (Table 1) and eight studies were conducted in humans (Table 2).

**Table 1:** Summary of animal studies

First author surname	Year of publication	Animal model	Results
Alizadeh	2015	Layer-type chicken	Significant dose-dependent effect of 2-AG injections (0.25 µg, 0.5 µg, 1 µg) on mean food intake (*p* < 0.01). Significant effect of 6.25 µg SR141716A injections on increased mean food intake (*p* < 0.01) but no effect at 12.5 µg and 25 µg
Brissard	2018	Mice	CB1R-/- knock out mice had significantly reduced preference for solutions containing rapeseed oil (*p* < 0.01) and linoleic acid (*p* < 0.003) compared to wild type mice
Brown	2018	Mice	OEA enhanced GLP-1 signalling through increased GLP-1 mediated cAMP production. Combination of Ex4 + OEA led to significantly greater weight loss (− 6.0 g ± 0.4 g) compared to Ex4 alone (− 4.6 g ± 0.4 g) or OEA alone (− 3.5 ± 0.2 g) (*p* < 0.0001). OEA had no significant effect on the hypophagic effects of GLP-1 and Ex4
Cottone	2009	Goldfish	Starvation for 24 h, 48 h and 8 days resulted in significant 1.4, 1.6 and 1.7 (respectively) fold increase in CNR1 concentration (*p* < 0.01) compared to fed goldfish. In 24-h food-deprived goldfish, AEA administration resulted in 1.2-fold lower CNR1 concentration compared to food-deprived fish not administered AEA
de Fonseca	2001	Rats	OEA administration had a dose-dependent nonsignificant effect on reducing food intake. Food intake increased over time after administration of OEA but did not reach the levels of control vehicle. SR141716A and SR144528 did not impact hypophagic effect of OEA
Deshmukh	2012	Rats	Both 2-AG and noladin ether significantly increased food intake (*p* < 0.001), which was attenuated by AM 251 pre-treatment. Both 2-AG and noladin ether significant increased preference for high-fat diets, even when in rats who had a natural preference for high-carbohydrate diets (based on a free-feeding experiment). Pre-treatment with AM 251 antagonised this effect
Droste	2010	Rats	AM 251 significantly (*p* < 0.001) reduced response to chocolate-flavoured pellets, with significant reductions at doses of 0.3 mg/kg (*p* < 0.05), 1.0 mg/kg (*p* < 0.05) and 3.0 mg/kg (*p* < 0.001). AM 251 did not alter response to normal grain pellets
Escartin-Perez	2009	Mice	ACEA injections resulted in significantly increased preference for carbohydrates (*p* < 0.0001) while protein and fat intake did not change. Effect was reversed by pre-treatment with AM 251
Fu	2008	Rats	Higher NAPE-phospholipase D expression results in longer time between last and first meals of the day (significant on days 8–10 after injection with adenoviral vector) and longer post meal intervals (significant on days 8–10 after injection)
Gardner	2006	Rats	Injections of O-2050 (0.03–3.0 mg/kg) and SR141716A (3.0 mg/kg) resulted in significantly reduced food consumption at hour 1 post-injection (for both O-2050 and SR141716A) and hour 3 (for O-2050 only). Injections did not result in significant weight loss 24 h after injection
Gianessi	2019	Rats	AM404 significantly reduced normal responses to food (*p* < 0.001) which was partially reversed by increased 2-AG
Gomez	2002	Rats	Twenty-four-hour food deprivation resulted in sevenfold increase in AEA and SR141716A administration resulted in a dose-dependent reduction in food intake in both 24-h fasted and partially satiated rats
He	2021	*Drosophila melanogaster*	Flies had a significantly higher preference for food with higher concentration (0.1 mg/ml vs. 0.01 mg/ml) of AEA (consistently) and 2-AG (for up to 4 days)
Higuchi	2010	Mice	O-2050 significantly reduced high-fat diet preference (*p* < 0.05) compared to a normal diet
Higuchi	2011	Mice	2-AG hypothalamic levels significantly increased after 3 days of a high-fat butter-based diet as compared to a standard soy-based diet. O-2050 administration significantly reduced preference for a high-fat diet
Higuchi	2012	Mice	2-AG levels were significantly higher after a conditioned place preference test (to assess preference with an environment previously associated with a high-fat diet) than before. This change was not detected in rats consuming a standard diet. O-2050 administration could suppress high-fat diet preference when administered throughout 14 days as well
Keyshams	2016	Chicks	2-AG injections (5.28 nmol) significantly increased mean food intake (*p* < 0.001)
Kirkham	2002	Rats	Administration of 0.5 and 2 µg of 2-AG resulted in significantly increased food intake and deprivation resulted in twofold higher AEA and threefold higher 2-AG levels, and 2-AG levels returned to control values when satiated again
Mahler	2007	Rats	AEA injections resulted 130–210% increased preference for sucrose, increased eating bouts by 203% and increased intake by 600%, as well as increased time spent eating by 254%
McLaughlin	2003	Rats	SR141716A and AM251 had a significant (*p* < 0.001), dose-dependent effect on reducing lever-pressing which lasted 15 and 22 h, respectively
Oveisi	2004	Rats	OEA administration in either gavage or capsule for resulted in significantly reduced feeding (*p* < 0.01 and *p* < 0.001, respectively). OEA did not have an effect on meal size but decreased the number of meals consumed and time between meals
Overton	2006	Rats	Observation of OEA binding to GPR119 through fluorescence. OEA administration resulted in significantly reduced 1 to 2 h after administration
Parker	2015	Rats	Combined effect of high-dose DAMGO and subthreshold dose of 2-AG resulted in significantly higher observations of food-seeking behaviour and food-related locomotor activity when compared to high DAMGO administration alone. Overall amount of food consumed did not differ
Provensi	2014	Mice	Histidine decarboxylase knock-out mice consumed similar amount of food than wild type mice and anorectic effect of OEA was significantly reduced in knock out mice (*p* < 0.01) at 45 and 60 min following administration
Pucci	2019	Mice	FAAH gene expression was significantly (*p* < 0.001) lower in brains of rats with binge-eating behaviour exposed to restriction and stress
Reyes-Cabello	2012	Rats	AEA did not have an effect on food-deprived rats but led to hyperphagia in partially satiated rats. AM404 had anorectic effects in food-deprived and satiated rats but resulted in increased intake when paired with GW6471. This effect was suppressed by the administration of SR141716A
Salaya-Velasquez	2020	Rats	Administration of AEA resulted in significantly higher (*p* < 0.0001) number of ΔFosB neurons in the nucleus accumbens
Soria-Gomez	2014	Mice	CNR1 receptors in the olfactory bulb enhance odour during hunger and increase food intake
Thabuis	2010	Rats	Food intake was 6.5% lower in mice consuming OEA compared to control. OEA feeding resulted in upregulation of GPR119 and FAAH
Thomson	2016	Rats	AM6527 administration at lower dose (0.6 mg·kg) resulted in behaviour similar to food reward value, while at higher doses (1.0 and 4.0 mg/kg) it resulted in behaviour pattern similar to satiety

**Table 2:** Summary of human studies

First author surname	Year of publication	Study design	Sample size	Population	Age	Results
Caruso	2012	Cohort	118	60 males, 58 females	65 and over	The rs1049353 genotype was significantly associated with higher odds of increased complex carbohydrate intake and decreased odds of higher intake of cholesterol and saturated fats, after controlling for age, gender and BMI
de Luis	2013	Cross-sectional	258	Obese individuals (mean BMI 36.3 ± 5.1), 64 males and 194 females	Mean age 48.1 ± 15.8	Individuals assigned a diet high in mono- or poly-unsaturated fatty acids did not have significant differences in weight, BMI, waist circumference, fat mass or systolic blood pressure regardless of presence of the rs1049353 genotype
Monteleone	2016	Cross-sectional	14	Obese individuals, 9 women and 5 men	23 to 55	2-AG levels increased before eating favourite food and decreased after eating favourite food in already satiated patients. 2-AG levels were significantly higher before eating favourite food than when not eating favourite food. AEA levels decreased during intake of non-favourite food and increased while eating favourite food
Monteleone	2017	Cross-sectional	7	Obese individuals with binge-eating disorder	23–55	AEA levels increased after eating preferred food but decreased after eating non-preferred foods. No change on 2-AG levels was seen
Monteleone	2012	Cross-sectional	8	3 men, 5 women, normal eating behaviours	21–33	2-AG levels were significantly higher before and during intake of preferred foods when compared to eating non-preferred foods and that 2-AG levels were significantly positively correlated with plasma ghrelin levels
Monteleone	2009	Cross-sectional	462	134 patients with anorexia nervosa, 180 patients with bulimia nervosa and 148 normal weight	27.1 ± 7.2 (normal weight), 24.3 ± 6.1 (anorexia nervosa), 27.4 ± 6.6 (bulimia nervosa)	The rs324420 genotype was significantly more frequent among patients with anorexia and bulimia nervosa
Sipe	2005	Cross-sectional	2667	46.4% male	57.2 ± 14.1	The rs324420 genotype was significantly associated with overweight and obesity in participants on white and black ancestry but not among participants with Asian ancestry
Tomassini	2013	Cross-sectional	17	Not specified	27.58 ± 1.16	In non-PROP tasters, higher OEA levels were significantly correlated with lower perceived hunger and higher plasma AEA levels were correlated with higher restraint and lower perceived hunger. Plasma AEA and 2-AG levels were significantly lower in non-tasters

### Endocannabinoid Compounds and Eating Behaviour

Various studies have previously investigated the link between ECs and food intake and preferences in both animal models and humans. In a recent study, researchers presented *Drosophila melanogaster* with foods containing ECs, including AEA, 2-AG, 2-linoleoyl glycerol (2-LG) and arachidonic acid (AA), as well as phytocannabinoids (from *Cannabis sativa*) and synthetic cannabinoids (mainly formulated for pharmaceutical purposes) [12]. Liquid food consumption containing sucrose and yeast with cannabinoids was compared to control liquid feed. Authors found that the flies had a significantly stronger preference for food containing higher concentrations of 2-AG (for up to 4 days) and AEA (sustained throughout experiment) [12]. Foods containing phytocannabinoids and certain synthetic cannabinoids were also preferred. Interestingly, the authors also found that AEA and 2-AG suppressed food intake, and that AEA significantly increases survival rate in starving fruit flies, potentially by reducing lipid metabolism as demonstrated by higher levels of triglycerides in flies that previously consumed EC-containing foods [12]. The main caveat to consider with this study, as explained by the authors, is that CNR1 and CNR2 are not expressed in *Drosophila*; thus, the effects of ECs observed may not be applicable in humans or other animal models that express these receptors. However, this may provide some clues into reactions to ECs in reduced expression of CNR1 and CNR2.

A previous study found an inverse association between ECs and hedonic eating in humans. Normal weight, healthy subjects that had reached satiation were given palatable foods of their preference (i.e. traditional cakes) that they could consume ad libitum in an initial session which was followed by a second session where they were provided the same amount of non-preferred isocaloric foods similar in nutritional value (i.e. bread, milk, butter) that could also be consumed ad libitum and combined as desired (i.e. bread and butter, bread and milk) [13]. It was found that 2-AG levels were significantly higher before and during hedonic eating of preferred foods when compared to eating non-preferred foods and that 2-AG levels were significantly positively correlated with plasma ghrelin levels [13]. This has also been tested in subjects with binge-eating disorder (BED) and obesity. In subjects with BED, 2-AG levels were not significantly different between preferred and non-preferred foods, but rather AEA levels significantly increased after eating preferred foods, while in obese individuals 2-AG significantly increased before eating preferred foods and AEA levels decreased [14, 15].

There is also evidence of and interaction between the ENS and opioid system in rats. In a 2015 study, Parker et al. administered varying doses of u-opioid agonist D-Ala2, NMe-Phe4, Glyol5-enkephalin (DAMGO) and 2-AG in nucleus accumbens, involved in the hedonic food response, of Sprague–Dawley rats fed a high-fat diet [16]. The authors found that the combined effect of high-dose DAMGO and subthreshold dose of 2-AG resulted in significantly higher observations of food-seeking behaviour and food-related locomotor activity when compared to high DAMGO administration alone [16]. However, the effect on overall consumption (grams consumed) was nonsignificant, although there was a trend towards increased consumption [16]. Nevertheless, the authors conclude a potential significant interaction between the opioid and endocannabinoid system on high-fat feeding [16].

The hyperphagic effects of 2-AG have also been detected in layer-type chicken where administration of 2-AG resulted in a significantly higher food intake and SR141716A, a CNR1 antagonist, resulted in inhibited appetite [17, 18]. Additionally, another study also found that administration of AEA in Wistar rats provided with sucralose solutions resulted in a significantly higher expression of ΔFosB in the nucleus accumbens (compared to rats that were not administered AEA), which would also indicate a potential change in the food reward system due to the administration of AEA [19]. However, similarly to the latter study, the authors did not show any impact on the overall intake of sucralose in rats given AEA [19]. The importance of AEA in eating is supported by a study led in Wistar rats that were administered AM404, GW6471 and SR141716A, drugs known to interfere with metabolism of ECs [20]. In this study, administration of AM404 and PPAR-α antagonist, GW6471, led to increased food intake while SR141716A resulted in suppressed appetite and counteracted the appetite stimulating effects of AM404 [20]. This may suggest that AEA stimulates appetite by binding to CNR1 but when the activity of this receptor is inhibited, hunger is suppressed. This had also previously been demonstrated in a study wherein male Wistar rats were injected with AEA which stimulated food intake, while SR141716A inhibited food intake [21]. Additionally, OEA administration also increased feeding, with a possible synergistic effect with SR141716A [21]. The 2-AG compound may also be related to food preference via its interaction with CNR1 as shown in male mice fed a high-fat diet with resulted in increased 2-AG plasma levels and a subsequent increased preference for high-fat diet [22, 23].

There may also be a link between ECs and preference for sweet taste. In a study, Sprague–Dawley rats were injected with doses of AEA and a control vehicle in their nucleus accumbens 48 h apart to identify whether AEA had an impact on preference for sucrose, dislike of quinine (bitter taste) and overall food intake [24]. It was found that upon AEA administration sucrose preferences were up to two times higher than when control vehicle was administered, and that time spent eating and eating bouts were more than doubled, resulting in an overall sixfold increase in intake [24]. AEA had no effect on reactions to bitter taste [24]. There is also evidence suggesting that EC levels may vary by fed state. In one of the first studies to investigate EC variations in the brain, Kirkham et al. found that fasting led to higher anandamide and 2-AG levels in the brain of Lister hooded rats compared to rats fed ad libitum, while eating resulted in declining 2-AG levels [25•]. This study also further validates the findings of previous studies that 2-AG administration increases food intake while SR141716A has an anorectic effect.

Differential effects of ECs may occur depending on variation in individual taste sensitivity to 6-n-propylthiouracil (PROP). PROP taste sensitivity is associated with a detection of bitter taste in foods (or super tasters), while those who are not sensitive to PROP (or non-tasters) have been found to have a preference for high-fat, energy-dense foods [26]. In a study, individuals were groups by PROP sensitivity (super tasters or non-tasters) and were asked to complete a Three-Factor Eating Questionnaire as well as to provide a blood sample for a plasma EC profile [26]. Significant associations were found in non-tasters only where higher OEA plasma levels were significantly correlated with lower perceived hunger, while higher plasma AEA levels were correlated with higher restraint and lower perceived hunger [26]. Surprisingly, it was found that plasma AEA and 2-AG levels were significantly lower in non-tasters, although the inverse may have been expected due to the association with higher energy diets and previous studies presented here that have found higher AEA and 2-AG associated with hyperphagic effects [26]. In fact, a previous study has found that 2-AG, along with another endocannabinoid, noladin ether, is significantly associated with hyperphagia and increased high-fat feed consumption in rats as compared to high-carbohydrate feed [27]. Additionally, this study highlights that the effects of 2-AG and noladin ether on intake and diet preference can be antagonised by pre-treatment with AM 251 and that noladin ether could have a more potent effect on overall intake than 2-AG [27].

### Oleoylethanolamide and Eating Behaviour

Although EC-like compounds resemble ECs, they may have distinct effects on food intake. In particular, research has mainly focused on the effects of OEA. In fact, in free-fed male Wistar rats, OEA capsule administration resulted in appetite inhibition which persisted for over 24 h [28•]. Additionally, OEA may not only have an anorectic effect but it may also result in increased satiety as shown by increased time between meals in rats injected with an adenoviral vector causing them to produce higher amounts of NAPE-phospholipase D which catalyses the synthesis of OEA from NAPE [29]. This resulted in higher intestinal OEA production and increased satiety in rats, which was paired with higher expression of proliferator-activated receptors (PPAR-α) and CD36, both known to be involved in energy balance [29]. In a different rat model, OEA administration resulted in anorectic effects which were not inhibited by CNR1 and CNR2 antagonists SR141716A and SR144528 further reinforcing the notion that OEA does not interact with cannabinoid receptors to modulate eating behaviours [30], thus highlighting the need to look to interaction with other receptors and systems to understand how OEA relates to eating behaviour.

In fact, studies have put forward various potential mechanisms for this relationship. One such study was carried out in mice in which histidine decarboxylase gene (*HDC*), which codes for an enzyme responsible for the synthesis of histamine, was knocked out. Authors found that lack of histamine release, which was found to increase with OEA levels, resulted in attenuated anorectic effects of OEA [31], thus highlighting a close relationship. Additionally, there is evidence that OEA may enhance glucagon-like peptide 1 (GLP-1) signalling, involved in glucose homeostasis [32]. OEA may also interact with extendin-4 (Ex4), a GLP-1 receptor agonist, to enhance weight loss in obese mice in a synergistic manner, despite having no impact on the hypophagic effects of GLP-1 and Ex4 [32]. However, no effect of OEA on hypophagic it has also been elucidated that OEA may act by upregulating the OEA-specific G protein coupled receptor 119 (GPR119), which has been previously identified as a mediator in the anorectic effects of OEA, and FAAH, which is responsible for the degradation and cell uptake of ECs such as 2-AG which may increase food intake as previously discussed [33, 34].

### Cannabinoid Receptors, Fatty Acid Amide Hydrolase and Eating Behaviours

In both animal and human studies, cannabinoid receptors, particularly CNR1, and FAAH have been found to modulate food intake. In goldfish, it has been found that food deprivation leads to increased CNR1 expression in the brain, which is lowered upon refeeding or upon administration of AEA [35]. In rats, it has also been shown that CNR1 receptor antagonists, AM251, AM404, O-2050 and SR141716A, significantly suppress food intake behaviour regardless of macronutrient content of feed [36–38]. It has been posited that CNR1 antagonists may act through both inducing satiation and reducing reward mechanisms associated with food [39]. Conversely, it has also been suggested that AM251 may act to reduce response to palatable foods such as chocolate-flavoured feed in rats, rather than decrease overall intake [40]. On the other hand, CNR1 stimulation enhances food intake [38].

CNR1 may also be involved in fat taste perception as a previous study demonstrated a reduced preference for fat among *CNR1* knockout mice [41]. This is also supported in a different study where administration of O-2050, a CNR1 antagonist, also resulted in reduced dietary fat preference in mice which had previously been fed a high-fat diet for 2 weeks and had developed a preference for this diet type [42]. However, a study in Wistar rats has also shown that activation of CNR1 via administration of CNR1 agonist N-[2]-5Z,8Z,11Z,14Z-eicosatetraenamide (ACEA) resulted in increased preference for carbohydrates when rats were also offered protein and fat feed [43]. This effect was counteracted by the administration of the CNR1 antagonist AM251, similarly to what was observed in Deshmukh and Sharma [27]. ACEA also reversed satiation in rats and led to increased feed time [43]. One study also alluded to a potential role of CNR1 in increasing odour detection which may be linked to increased food intake [44].

Furthermore, FAAH has been associated with certain eating patterns. For example, it has been previously shown that FAAH levels decrease in the brains of rats with binge-eating behaviours [45]. This has also been elucidated to in humans wherein higher frequencies of the FAAH 385 C to A single nucleotide polymorphism (SNP) (hereafter rs324420) have been associated with anorexia and bulimia nervosa [46]. Similar associations have also been seen for the CNR1 1359 G to A SNP (hereafter rs1049353) [46]. Additionally, rs324420 has also been associated with increase rates of overweight and obesity in a white and black populations, but not in an Asian population [47]. The rs1049353 SNP has also been associated with increased consumptions of complex carbohydrates and decreased dietary cholesterol and saturated fat [48•]. Despite this, rs1049353 was not found to be associated with weight loss [49].

## Conclusion

The literature reviewed in this report demonstrates that there is a clear link between ENS and eating behaviours which has been largely investigated in animal models. Overall, studies seem to indicate that 2-AG and AEA via CNR1 binding may stimulate hunger and food intake while OEA may inhibit hunger. Mechanisms of these associations are not yet well understood, although literature indicates that there may be interactions with other physiological systems to consider. Caruso et al. reported an association between rs1049353 and food group consumption [48•]. The association between rs1049353 and alcohol dependence has previously been studied in a meta-analysis, with no significant associations either [50]. Thus, it is important to continue to investigate this association in different populations given the known importance of FAAH in regulating food intake.

Given the paucity in human studies in this research area, future studies should focus on observing these associations in humans, specifically on whether variation in ENS genes can help explain differences in diet quality, hedonic eating and food intake. Although there appears to be a large pharmaceutical interest in this area to identify substances that target the ENS, such as the CNR1 antagonist rimonabant, and can result in suppressed appetite and weight loss, it is important to also explore the most appropriate diet therapies that consider the role of the ENS in eating behaviour. An additional aspect that this report did not consider is the role of exposures in the food environment, which has become increasingly obesogenic. Ubiquitous cues towards highly palatable foods may impact processes in the ENS and is an area for future research.
